# Editorial: The evolution in pharmacology of infectious diseases: 2025

**DOI:** 10.3389/fphar.2026.1907819

**Published:** 2026-07-10

**Authors:** Exequiel O. J. Porta, Ali Saffaei, Karunakaran Kalesh, Katrien Van Bocxlaer

**Affiliations:** 1 UCL School of Pharmacy, University College London, London, United Kingdom; 2 Department of Pharmaceutical Care, Sepid Nikan Hospital, Tehran, Iran; 3 School of Health and Life Sciences, Teesside University, Middlesbrough, United Kingdom; 4 National Horizons Centre, Darlington, United Kingdom; 5 Skin Research Centre, Hull York Medical School, York Biomedical Research Institute, University of York, York, United Kingdom

**Keywords:** antimicrobial resistance, drug repurposing, infectious disease pharmacology, neglected tropical diseases, phage therapy, pharmacokinetics/pharmacodynamics, precision medicine, therapeutic drug monitoring

## 1 Introduction

Infectious disease pharmacology continues to evolve at the intersection of therapeutic innovation, antimicrobial resistance, clinical optimization, and global health implementation. In this changing landscape, the field is increasingly required to connect mechanistic insight with clinically actionable, scalable, and globally relevant therapeutic strategies. The years following the acute phase of the COVID-19 pandemic have reinforced that pharmacological progress cannot be defined only by the discovery of new antimicrobial agents (Porta et al., 2024a). It also depends on understanding resistance at molecular and population levels, optimizing the use of existing therapies, developing regulatory and translational pathways for novel modalities, and ensuring that advances are relevant to neglected and difficult-to-treat infections.

## 2 The evolution in pharmacology of infectious diseases: 2025

The present Research Topic builds on the 2022 edition of “*The evolution in pharmacology of infectious diseases”*, which captured a period shaped strongly by COVID-19, real-world therapeutic evidence, and antimicrobial optimization (Porta et al., 2024b). The 2025 Research Topic extends that trajectory. Rather than centering on a single pandemic pathogen, it reflects a broader and more mature landscape in which infectious disease pharmacology is increasingly guided by resistance biology, pharmacokinetic/pharmacodynamic reasoning, therapeutic drug monitoring, host-directed approaches, chemoproteomic target discovery, and regulatory preparedness. Bringing together 10 articles from 76 researchers and attracting more than 100,000 total views and downloads at the time of writing (June 2026), this edition reflects sustained interest in infectious disease pharmacology as a multidisciplinary and globally relevant field.

A major theme emerging from this Research Topic is the need to understand antimicrobial resistance as a dynamic biological and translational challenge. Belay et al. reviewed major antibacterial resistance mechanisms, including target modification, reduced drug accumulation, enzymatic inactivation, biofilm formation, and horizontal gene transfer, while also emphasizing stewardship and next-generation antimicrobial strategies. This broad framework is complemented by Zhang et al., who examined drug-resistant *Mycobacterium tuberculosis* as a multifactorial problem involving genetic resistance, cell wall remodeling, metabolic adaptation, epigenetic regulation, and immune evasion. By connecting resistance mechanisms with the limitations of *Bacillus* Calmette–Guérin (BCG) and the promise of next-generation vaccine platforms, their contribution highlights the importance of integrating antimicrobial therapy with immunological prevention.

The Research Topic also advances conceptual thinking on resistance. Baquero et al. introduced antechokinetics as a framework for considering the intracellular kinetics of antimicrobial resistance molecules. By shifting attention from the movement of drugs alone to the movement, bioavailability, metabolism, and excretion of resistance biomolecules inside bacterial cells, this article opens a stimulating pharmacological perspective on how antibiotic action and resistance reactions may be studied together. At the translational and regulatory level, Fukaya-Shiba et al. addressed the development of phage therapy medicinal products for antimicrobial-resistant bacterial infections. Their analysis of quality control, manufacturing, non-clinical evaluation, clinical trial design, and Japan-specific regulatory considerations illustrates that innovation against resistance requires not only biological plausibility but also robust pathways toward standardized, safe, and timely patient access.

A second theme is the optimization of existing anti-infective therapies through clinical pharmacology. Liu et al. integrated global *Cryptococcus* minimum inhibitory concentration distributions with pharmacokinetic/pharmacodynamic parameters for key antifungals, providing clinically relevant insights into induction, consolidation, and maintenance strategies for cryptococcal meningitis and meningoencephalitis. Tan et al. further emphasized the value of individualized antifungal management through a real-world, age-stratified analysis of isavuconazole therapeutic drug monitoring. Their findings suggest that pediatric patients, and selected elderly patients with factors such as intensive care admission or renal replacement therapy, may particularly benefit from exposure-guided dosing. Together, these studies underscore that antifungal pharmacology is moving toward a more nuanced model in which susceptibility data, tissue penetration, patient characteristics, and measured drug exposure are integrated into therapeutic decisions. This patient-centered approach is also evident in the review by Spanakis et al. on drug-drug interactions between antiretroviral therapy and cardiovascular drugs. As people living with HIV experience longer life expectancy and a growing burden of cardiovascular comorbidity, therapeutic success increasingly depends on managing complex polypharmacy. Their analysis highlights how pharmacological tools, interaction databases, and clinical judgment must converge to prevent adverse outcomes while preserving antiviral efficacy and cardiovascular protection.

The third theme concerns therapeutic innovation for viral, neurological, and neglected tropical diseases. Bobat et al. reviewed the broad-spectrum therapeutic potential of 4-phenylbutyrate, a chemical chaperone that modulates endoplasmic reticulum stress and has been explored in ocular herpes simplex virus type 1 infection, HSV-1-associated neurological disease, and several systemic disorders. This contribution illustrates the continued relevance of drug repurposing and host-directed pharmacology, particularly where modulation of cellular stress pathways may provide benefit across infectious and non-infectious disease contexts. Additionally, two contributions focus on leishmaniasis, reinforcing the importance of neglected tropical diseases within the evolution of infectious disease pharmacology. Khamesipour et al. investigated the susceptibility of clinical *Leishmania* isolates to oleylphosphocholine compared with miltefosine and other standard antileishmanial agents, providing a clinically grounded assessment using *ex vivo* isolates. Porta et al. used activity-based chemoproteomic profiling with cell-permeable ATP-site probes to map the active kinome of *Leishmania mexicana*, identifying ligandable protein and metabolic kinases that may support future target prioritization. Together, these studies connect target discovery with translational drug evaluation and demonstrate how methodological innovation can inform the development of next-generation antileishmanial strategies.

Across the Research Topic, infectious disease pharmacology is presented as a field that must operate across scales: from intracellular resistance molecules to patient-specific dosing, from parasite chemoproteomics to clinical isolate susceptibility, and from promising modalities such as phage therapy to the regulatory systems required for implementation. The articles collectively show that future progress will depend on combining mechanistic depth with real-world clinical evidence, and on aligning drug discovery, pharmacometrics, stewardship, diagnostics, and policy.

## 3 Conclusion

In conclusion, “*The evolution in pharmacology of infectious diseases: 2025*” reflects a field moving beyond reactive therapeutic development toward integrated, anticipatory, and precision-informed strategies. The Research Topic highlights resistance as both a molecular and systems-level challenge, while emphasizing that innovation must be matched by optimized use, translational feasibility, and equitable relevance across global infectious disease priorities. Looking ahead, emerging approaches such as spatial analytical biology and high-resolution subcellular localization technologies may further expand infectious disease pharmacology by placing host-pathogen interactions, drug action, and therapeutic response within their precise cellular and tissue contexts. As infectious threats continue to change, pharmacology will remain central to transforming biological insight into safer, more effective, and more accessible interventions.
